# Managing Workload and Patient Flow in a Community Mental Health Team: A Prospective Quality Improvement Initiative

**DOI:** 10.1192/j.eurpsy.2026.10971

**Published:** 2026-07-31

**Authors:** G. Murtaza

**Affiliations:** 1 Child & Adolescent Psychiatry, Children’s Health Ireland; 2 Psychiatry, North Dublin Mental Health Services, Dublin, Ireland

## Abstract

**Introduction:**

Community Mental Health Teams (CMHTs)manage individuals with complex psychiatric needs in resource-limited settings. Structured approaches to referral triage, discharge planning, and follow-up of non-engagement are key to ensuring equitable access and safe caseloads. This quality improvement project aimed to optimise patient flow and improve service efficiency.

**Objectives:**

To evaluate caseload activity in a CMHT over six months, focusing on discharges, non-engagement, and new referrals.

**Methods:**

This prospective QI initiative was conducted at CMHT Swords – Orange Team, North Dublin Mental Health Services, between January 13 and July 13, 2025. A total of 131 existing patients and 20 new assessments were reviewed. Outcomes were categorised by discharge type, engagement status, or administrative transfers and reviewed through MDT discussions and GP correspondence.

**Results:**

Out of 151 total patients, 48 (31.8%) exited CMHT care during the audit period. This included 19 (12.6%) discharged due to persistent non-attendance or disengagement — most with EUPD, EUPD traits, or mild anxiety/depression, and none deemed high-risk. Additionally, 4 patients (2.6%) were discharged stable to primary care, 2 (1.3%) due to behavioural non-engagement, 5 (3.3%) were transferred following relocation, and 1 (0.7%) was referred for high-support residential care. Of 20 new assessments, 16 (10.6%) were discharged with GP follow-up advice, and 4 (2.6%) were linked for ongoing care.

**Image 1:**
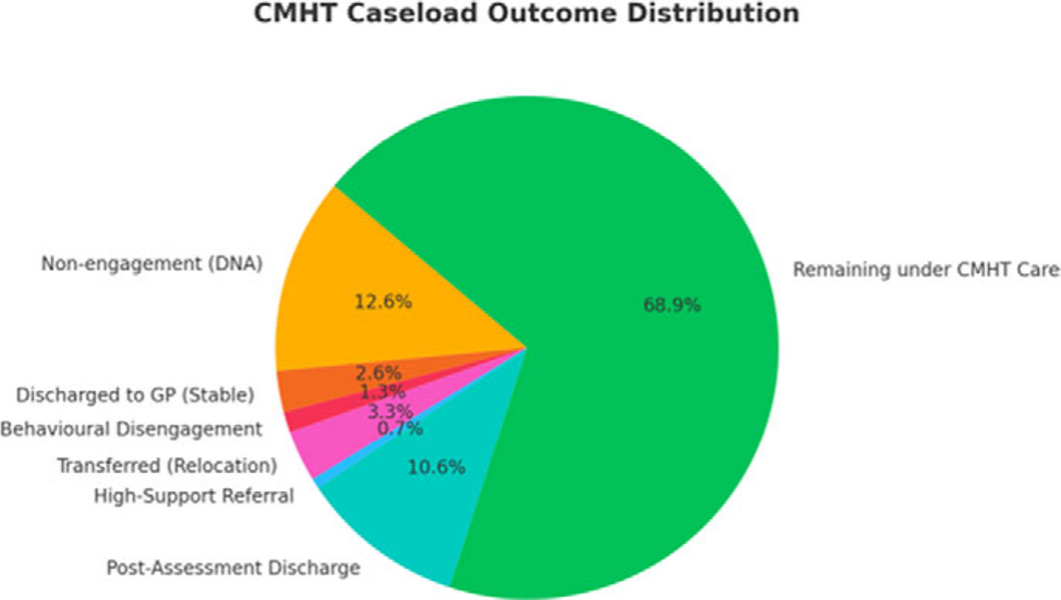

**Image 2:**
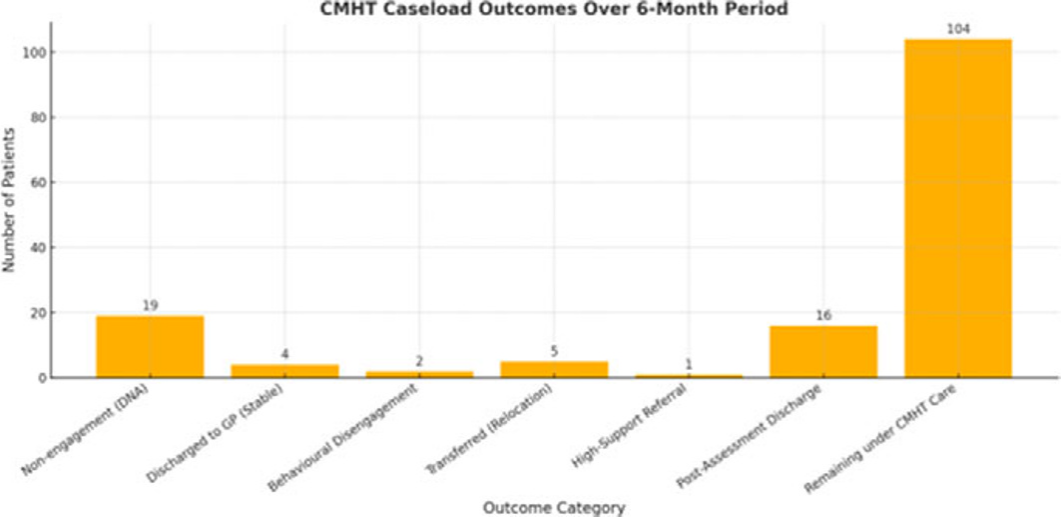
Image 2: Long description.

**Conclusions:**

Structured monitoring of caseloads, including safe discharge planning and triage of new referrals, supported improved team capacity and patient flow. The findings highlight how a focused QI approach can enhance service accessibility while maintaining safe and person-centred care within a CMHT setting.

**Disclosure of Interest:**

None Declared

## Long descriptions

### Image 2: Long description

The y-axis is labeled Number of Patients and ranges from 0 to 100 in increments of 20. The x-axis is labeled Outcome Category. The chart displays seven orange bars with the following data points from left to right.

* Non-engagement D N A: 19 patients.

* Discharged to G P Stable: 4 patients.

* Behavioural Disengagement: 2 patients.

* Transferred Relocation: 5 patients.

* High-Support Referral: 1 patient.

* Post-Assessment Discharge: 16 patients.

* Remaining under C M H T Care: 104 patients.

The final bar representing patients remaining under care is significantly higher than all other categories combined.

Navigate back to Image 2:.

